# Correlation between the spatial distribution and colony size was common for monogenetic bacteria in laboratory conditions

**DOI:** 10.1186/s12866-021-02180-8

**Published:** 2021-04-15

**Authors:** Heng Xue, Masaomi Kurokawa, Bei-Wen Ying

**Affiliations:** School of Life and Environmental Sciences, University of Tsukuba, 1-1-1 Tennoudai, Tsukuba, Ibaraki, 305-8572 Japan

**Keywords:** Spatial distribution, Colony size, *Voronoi* diagram, Population growth, Experimental ecology

## Abstract

**Background:**

Geographically separated population growth of microbes is a common phenomenon in microbial ecology. Colonies are representative of the morphological characteristics of this structured population growth. Pattern formation by single colonies has been intensively studied, whereas the spatial distribution of colonies is poorly investigated.

**Results:**

The present study describes a first trial to address the questions of whether and how the spatial distribution of colonies determines the final colony size using the model microorganism *Escherichia coli*, colonies of which can be grown under well-controlled laboratory conditions. A computational tool for image processing was developed to evaluate colony density, colony size and size variation, and the *Voronoi* diagram was applied for spatial analysis of colonies with identical space resources. A positive correlation between the final colony size and the *Voronoi* area was commonly identified, independent of genomic and nutritional differences, which disturbed the colony size and size variation.

**Conclusions:**

This novel finding of a universal correlation between the spatial distribution and colony size not only indicated the fair distribution of spatial resources for monogenetic colonies growing with identical space resources but also indicated that the initial localization of the microbial colonies decided by chance determined the fate of the subsequent population growth. This study provides a valuable example for quantitative analysis of the complex microbial ecosystems by means of experimental ecology.

**Supplementary Information:**

The online version contains supplementary material available at 10.1186/s12866-021-02180-8.

## Background

As an ancient finding in microorganisms, the colony is the representative morphological characteristic shared by most asexual microbes. Colony formation results in a structured population and is considered a survival strategy allowing bacteria [1] to adapt to environmental changes [2] and to develop resistance to antibiotics [3, 4]. Investigation of microbial colonies is crucial to achieve a fundamental understanding of population growth in microbial ecology [5]. To date, mainstream research on bacterial growth has been conducted in liquid media, where all cells tend to obtain resources equally, and the interactions are generally uniform. In nature, microbes often inhabit a solid environment [6], which results in a large variation in population size, regardless of the genetic and environmental conditions. As the mechanisms of population growth in liquid are not always true or applicable for colony growth, the growth dynamics of single colonies in well-defined experimental conditions, e.g., on agar plates, have been studied widely by various approaches. The diffusion and pattern formation of single colonies were found to follow physical principles [7–9]. The mechanisms underlying colony growth dynamics are often explained by chemical interactions [10] and substrate diffusion in the medium [11]. Pattern formation of a single colony is known to be mediated by mechanical interactions [12]. In particular, recent studies have demonstrated that mechanical interaction plays an important role in determining the sizes of monogenetic colonies [13] and the patterns of polygenetic colonies [14].

On the other hand, the size variation of colonies has rarely been studied under laboratory conditions, although it has been observed not only in genetically differentiated microbial communities but also in monogenetic bacterial populations. However, the colony is used as a basic form in microbiological experiments. Although size variation of colonies on the same medium surface (space) is recognized as a common feature, quantitative analysis of the size-differentiated colonies has not been appropriately conducted. The spatial distribution of colonies, which is a fundamental aspect of soil bacteria in nature [15, 16], is thought to contribute to size variation. The first study connecting spatial distribution to size variation was recently reported; this study examined bacterial colonies in well-defined experimental conditions [17]. A quantitative association between colony size and spatial distribution was observed under limited experimental conditions. Whether and how genetic or environmental changes affect the relationship between the spatial distribution of colonies and size variation remain unclear.

To address this question, the present study used laboratory strains of *Escherichia coli* (*E. coli*) with different genomes and well-defined culture conditions with different nutritional levels. *E. coli* has been applied as a powerful model microorganism for studies ranging from molecular analyses to population-level analyses [18] and is commonly used in experimental evolution [19, 20]. *E. coli* colonies growing on agar media can suitably mimic the ecosystem of microbes living on solid surfaces. Our previous studies experimentally and theoretically demonstrated the coordination of genome reduction and population growth in liquid media [21, 22], as well as changes in growth due to nutritional conditions [22, 23]. The reported *E. coli* strains and the corresponding media were adopted in the present study to investigate whether and how genome reduction and nutritional decline caused any changes in colony size variation and the rule of spatial distribution.

## Results

### Experimental and computational approaches

**Fig. 1:**
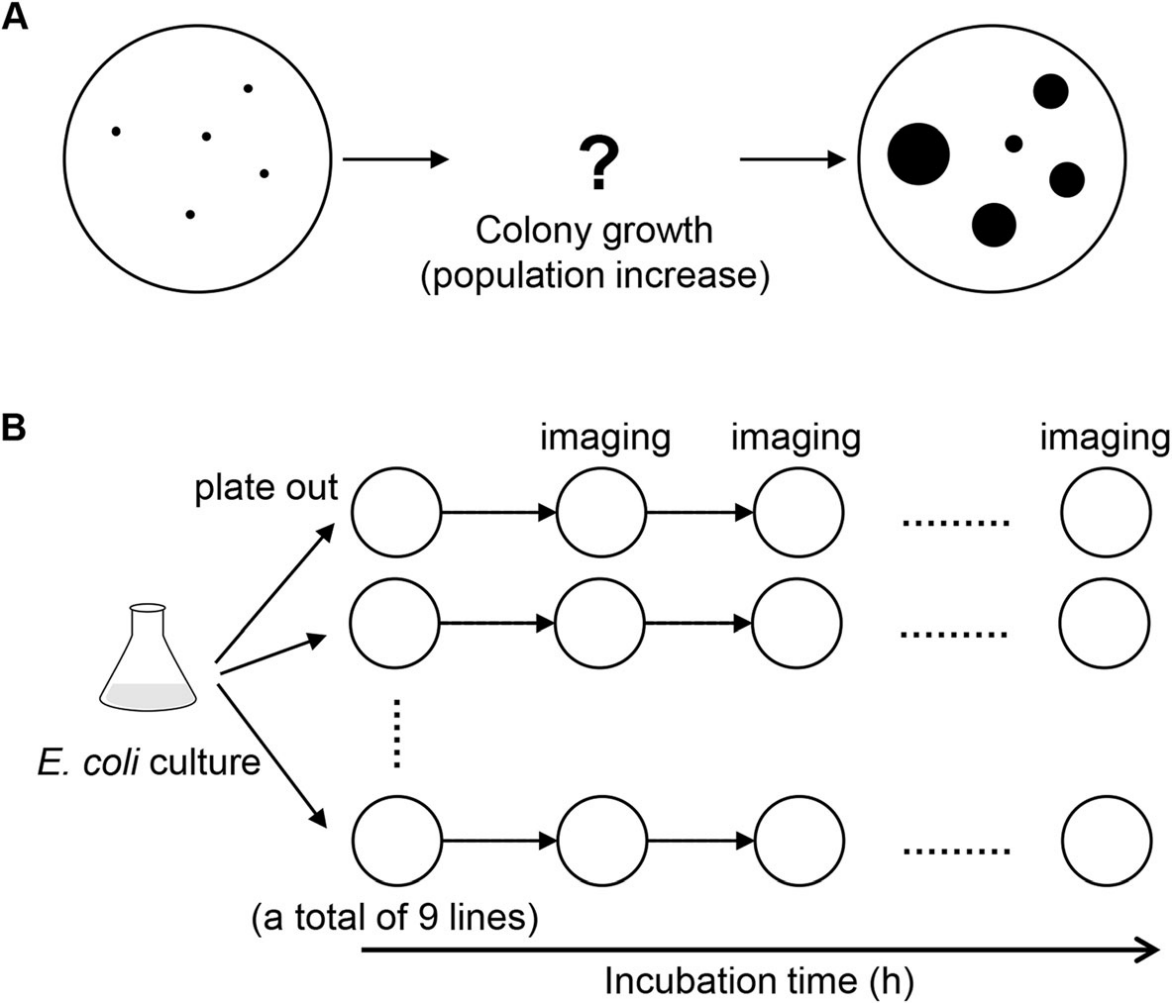
Experimental ecology. **a.** Schematic drawing of the question addressed in the present study. **b.** Illustration of the experimental scheme. Nine replicates (agar plates) were performed for each condition

Laboratory strains of *E. coli* were used to investigate the relationship between the spatial distribution and increase in population (Fig. 1a). The *E. coli* cell cultures were diluted and plated on agar plates (Fig. 1b), and the single colonies formed on the agar plates were considered geographically separated populations. The colony size is approximately equivalent to the population size in a certain environment. As the mean size of the colonies formed on the same agar plate is thought to be decided by both the genome and the environment, genomic and nutritional differences were introduced in the experiments. Both complete (LB) and minimal (M63) media (agar plates) were applied to mimic rich and poor nutritional conditions, and wild-type and genome-reduced *E. coli* strains were used. A total of nine replicates were performed for each condition, and the temporal changes in colony growth were recorded by a CCD camera (Fig. 1b). The recorded images (photos) were subjected to image processing with a new program developed in the present study.

**Fig. 2:**
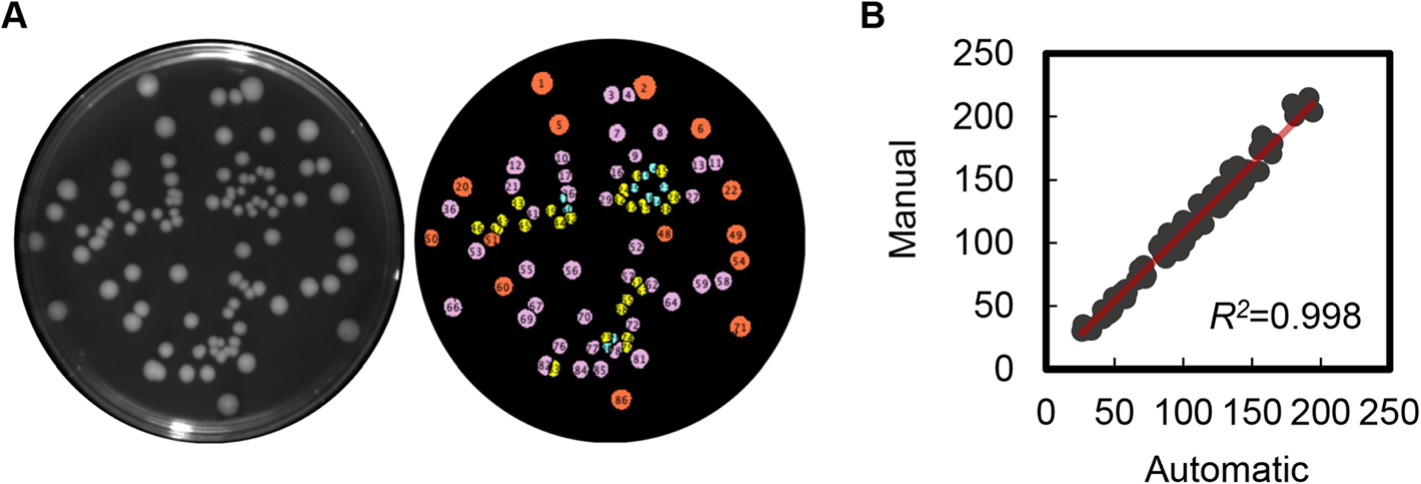
Colony count. **a.** Image data processing. The left image is a representative CCD camera photograph of the monogenetic *E. coli* colonies formed on the agar plate as an experimental result. The right image shows the colony count and evaluation with the newly developed computational program. **b.** Comparison of automatic and manual counts of colonies. The numbers of colonies formed on the agar plate were counted by eye (manual) and by the original Java script add-in program executed in Fiji software (automatic) and were plotted against each other. The linear regression is indicated by the red solid line, and its significance (*R*^*2*^) is shown

In addition, the automation of colony counting and image analysis was customized for efficient and quantitative evaluation of the connection of the spatial distribution with the colony/population size. A computational program implemented in ImageJ was developed for high-throughput image processing of the agar plates (Fig. 2a). For an example plate, number of colonies counted automatically with the original developed program was highly significantly correlated with the number obtained with manual counting (Fig. 2b). The slope of the correlation was ~ 1, indicating the equivalence of the two methods. This demonstrated that the image processing program was quantitatively reliable and applicable for colony analysis.

### Changes in the mean size and size variation of colonies caused by genomic and nutritional changes

**Fig. 3:**
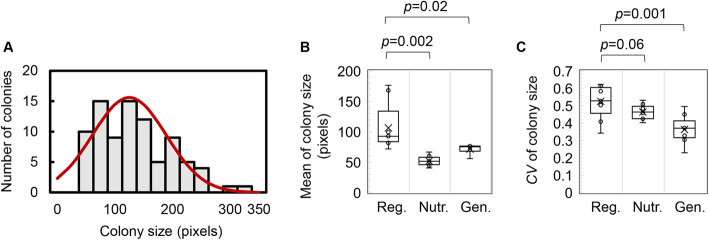
Colony size and size variation. **a.** Size distribution of the colonies on a single plate. The size differentiation of the *E. coli* colonies formed on a single LB agar plate is shown as an example. Fitting of the histogram to the Poisson distribution is indicated by the red curve. **b.** Boxplot of the mean values of the colony sizes. The sizes of the colonies formed on identical plates were evaluated, and the mean size of these colonies was calculated for each agar plate. The tiny circles and the crosses represent the mean sizes of colonies formed on the individual agar plates and the average of the mean sizes (on identical plates), respectively. Reg., Nutr. and Gen. indicate colony growth under regular conditions and in response to nutritional and genomic changes, respectively. The regular conditions and nutritional and genomic changes represent the wild-type strain growing on an LB agar plate, the wild-type strain growing on an M63 agar plate and the genome-reduced strain growing on an LB agar plate, respectively. **c.** Boxplot of the size variation of the colonies. The coefficient of variation (*CV*) of the colony size was calculated for each agar plate. The tiny circles and the crosses represent the *CV* of colony size on the individual agar plates and the average *CV*, respectively. Statistical significance is indicated. The data used for the boxplots could be found in Table S1

Whether the genomic and nutritional changes disturbed the mean size and size variation of colonies grown on the same agar plate was examined. As an example, the relative sizes of the colonies on a single agar plate were automatically determined (Fig. 3a), showing a mean colony size of approximately 110 pixels. The means of the nine replicates (Fig. 1b) are shown as boxplots (Fig. 3b). The mean size of the colonies decreased (*p* < 0.05) in response to either genomic or nutritional changes (Fig. 3b), although the colony densities on the plates were equivalent. The colonies carrying the reduced genome, in which ~ 21% of the genomic region was deleted from the wild-type genome [24], presented an ~ 30% decrease in colony size, from ~ 110 to 70 pixels. The decrease in colony size was more significant, ~ 50% compared to the wild-type colonies, as the growth condition changed from nutrient rich to nutrient poor. As the colony size here was evaluated within the stationary growth phase, close to the steady state, it represented roughly the maximal population size. The results indicated that the nutritional change affected the colony/population size more substantially than the genomic change did. This finding seemed to differ from that in liquid culture, which showed that the maximal OD_600_ was most strongly disturbed by genome reduction [22].

Size variation among colonies grown on the same agar plate was commonly found in all the conditions. For instance, the relative sizes of the 86 colonies on the same agar plate varied from ~ 50 to 300 pixels (Fig. 3a). This demonstrated that there was a large variation in colony size although these colonies had the same genome and grown under the same nutritional condition. The coefficient of variation (*CV*) was used to evaluate the size variation of the colonies on the same agar plate. In comparison to the regular conditions, both genome reduction and nutritional decline seem to decrease the size variation (Fig. 3c). In particular, the variation decreased highly significant due to the genome reduction (*p* = 0.001). This result suggested that the population increase of the monogenetic *E. coli* within the defined space was influenced by the genome more than by the nutritional condition of the space. Although the size variation of microbial colonies is frequently observed in laboratories, the present study verified such size variation of the monogenetic colonies distributed on the same plate and evaluated the genomic and nutritional contributions to the size variation in a quantitative manner for the first time.

### Correlation between colony size and the Voronoi area in common

**Fig. 4:**
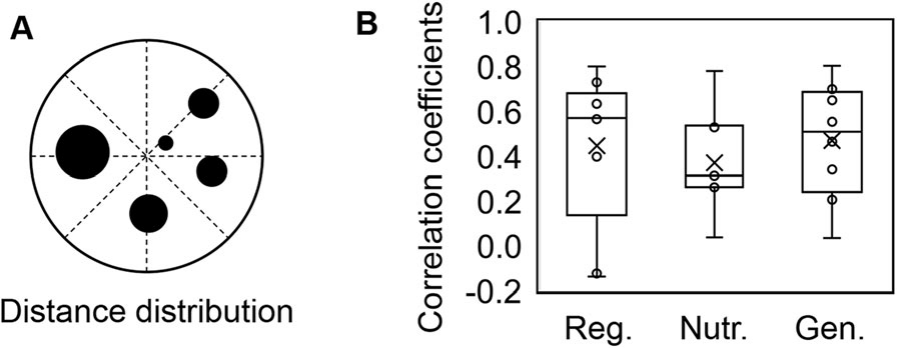
Spatial analysis of the distance from the centre. **a.** Illustration of the spatial analysis of the distance from the centre. The closed circles and the broken lines indicate the colonies and the direct distance from the centre of the plate, respectively. **b**. Boxplot of the correlation coefficients between the distance from the centre and the colony size. Reg., Nutr. and Gen. indicate colony growth under regular conditions and in response to nutritional and genomic changes, respectively. The regular conditions and nutritional and genomic changes represent the wild-type strain growing on an LB agar plate, the wild-type strain growing on an M63 agar plate and the genome-reduced strain growing on an LB agar plate, respectively. The data used for the boxplot could be found in Table S1

The reason for the size variation of the monogenetic colonies in the same space was investigated by spatial analysis approaches. First, whether the distance from the centre of the plate to the colony determined the colony size was analysed (Fig. 4a) because the colonies located at the centre of the plate seemed to be smaller than those at the edge of the plate (Fig. 2a). The correlation between colony size and the distance from the colony to the centre of the plate was evaluated. The results showed that colony size was weakly correlated with the distance from the centre of the plate, as the correlation coefficients showed large variations among the agar plates (Fig. 4b), indicating that the distance from the centre of the plate to the colony did not fully determine the colony size. Neither genome reduction nor nutritional decline changed the significance of the correlation (Fig. 4b). The results suggested that the colonies far away from the centre were subject to the edge effect to a great extent.

**Fig. 5:**
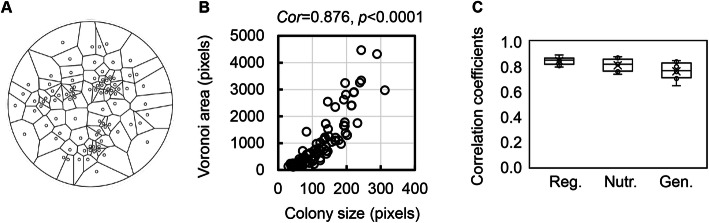
Spatial analysis by the *Voronoi* diagram. **a.** *Voronoi* diagram. An example of the *Voronoi* division of an agar plate according to the spatial distribution of the colonies is shown. The tiny open circles in the right image indicate the locations of the colonies, and the irregularly divided regions represent the calculated *Voronoi* areas corresponding to individual colonies. **b.** Scatter plot of the *Voronoi* area and colony size. As an example, the colony sizes are plotted against the respective *Voronoi* areas in a single agar plate. The correlation coefficient between the *Voronoi* area and the colony size (*cor*) and its statistical significance (*p*) are indicated. **c.** Boxplot of the correlation coefficients between the *Voronoi* area and colony size. The tiny circles and the crosses represent the correlation coefficients of individual agar plates and the average of the correlation coefficients, respectively. Reg., Nutr. and Gen. indicate colony growth under regular conditions and in response to nutritional and genomic changes, respectively. The regular conditions and nutritional and genomic changes represent the wild-type strain growing on an LB agar plate, the wild-type strain growing on an M63 agar plate and the genome-reduced strain growing on an LB agar plate, respectively. The data used for the boxplot could be found in Table S1

Alternatively, the spatial distribution of neighbouring colonies might play a crucial role. To verify this hypothesis, the two-dimensional space governed by the colony was subsequently evaluated. Spatial analysis techniques [25] were adopted to achieve a quantitative estimation when considering the plate as a metric space [26]. According to a previous report [17], the *Voronoi* diagram was applied to the analysis of the spatial distribution of colonies. Each colony on the plate was considered a mother point, and the plate was used as a metric space for *Voronoi* division. The *Voronoi* diagram successfully divided the space of the plate for the colonies, and the resultant separated regions were designated the *Voronoi* areas for the corresponding colonies (Fig. 5a). Intriguingly, the colony size and the *Voronoi* area were positively correlated in the same space/plate (Fig. 5b). A significant correlation (*p* < 0.001) was identified in all replicates and was common, regardless of the genomic and nutritional changes (Fig. 5c). This finding indicated that the correlation between colony size and the *Voronoi* area was universal, at least in the case of the laboratory *E. coli* strains. Further study is required to address whether the universality remained independent of the number of colonies per plate, which was somehow affected the colony size.

### Increase in the significance of correlation along with colony growth

**Fig. 6:**
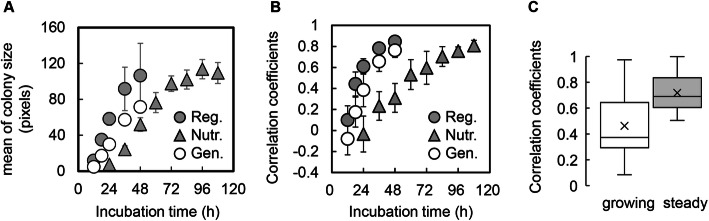
Temporal changes in the correlation coefficients between the *Voronoi* area and colony size. **a.** Temporal changes in the mean colony size. **b.** Temporal changes in the correlation coefficients between the *Voronoi* area and colony size. The analyses were performed with an interval of 12 h. Standard errors, representing the variation among the plates for each condition, are indicated. Reg., Nutr. and Gen. indicate colony growth under regular conditions and in response to nutritional and genomic changes, respectively. The regular conditions and nutritional and genomic changes represent the wild-type strain growing on an LB agar plate, the wild-type strain growing on an M63 agar plate and the genome-reduced strain growing on an LB agar plate, respectively. The data of the temporal changes used for the analysis was summarized in Table S2. **c.** Boxplot of the correlation coefficients between the *Voronoi* area and colony size. The open and shaded boxes indicate that the colonies of the other five different *E. coli* strains remained in the growing (1–2 days) and steady (1 week) phases, respectively. The crosses in the boxes represent the average correlation coefficients

Whether the growth phase of the colony contributed to the correlation was further analysed. Both genome reduction and nutritional decline cause a significant decrease in population growth in liquid culture [22, 27], and colony growth on solid medium also becomes slower. Whether the correlation between colony size and the *Voronoi* area changed with colony growth was analysed temporally in a 12-h interval (Fig. 1b). The growth of the colonies decreased in the genome-reduced and nutritionally poor conditions (Fig. 6a). The time required to reach the steady state of colony growth, i.e., the maximal population size, differed. The correlation coefficients between colony size and the *Voronoi* area increased significantly with colony growth (Fig. 6b). The colony growth-dependent increase in the strength of the correlation was a common feature, regardless of the variation in genomes and media. The results suggested that the correlation between colony size and the *Voronoi* area was significant only when the colony growth was close to the steady state. Additional experiments using the different genetically engineered *E. coli* strains showed that the positive correlation between colony size and the corresponding *Voronoi* area improved greatly in the steady phase in comparison to that in the growing phase (Fig. 6c), which clearly demonstrated the universality of the increase in the significance of correlation along with colony growth. This finding not only explained the disagreement between the present and previous studies, which was caused by the timing of colony growth reaching the steady state, but also indicated that the maximal population/colony size was connected to the spatial resource occupied by the colony (*Voronoi* area). To verify the assumption, additional investigation linking the changes in *Voronoi* areas to the growth dynamics of individual colonies, from the early exponential phase to the final saturated phase, is required.

In addition, the correlation coefficients gradually approached the maximal value of one, while the colony size was close to the maximum, which was commonly observed in all conditions (Fig. 6b). It indicated that space division occurred in a fair manner among the colonies, independent of genomic and nutritional differences. The initial localization of the colony (the first cell) determined the rule of spatial distribution for colony growth; consequently, the maximal colony/population size was determined.

## Discussion

Although the variation in *E. coli* colony size could be explained by *Voronoi* diagrams was previously reported [17], the results here further suggested that such phenomenon could be highly common independent of the media conditions and microbial genotypes. In addition, the temporal observation of colonies grown on the agar plates newly demonstrated that the predictive power of *Voronoi* diagrams increased associated with the population growth. The present study successfully found that such a positive correlation between colony size and *Voronoi* area appeared to be across the two different media and multiple *E. coli* strains. Nevertheless, whether this kind of spatial distribution could be considered as a null model for size variance between competing colonies required to be demonstrated by additional experimental assays with a large variety of both genetic and environmental conditions.

Note that the novel finding of the universality of the correlation between colony size and the *Voronoi* area was partially inconsistent with the previous study, which reported that it was only the wild-type *E. coli* strain grown on an LB plate that showed the positive correlation [17]. We assumed that the disagreement could be partially resulted from the varied experimental conditions. As the temporal changes in the correlation associated with colony growth were clearly detected in the present study (Fig. 6), the variation in the growth phase might be one of reasons of the difference in correlations. As the colonies of different species grown in varied conditions were all analysed in the same time scale, the slow-growing ones might have not reached the steady state and resulted in a poor correlation. Nevertheless, the difference in species might lead to the changes in correlation. Although the correlation was commonly detected in both the wild-type and the reduced genomes in the present study, the two genomes were derived from the same *E. coli* strain. We assumed that larger difference in genotypes could cause the changes in the correlation between colony size and the *Voronoi* area, due to the differentiated capacity of resource utilization. Further study on an assortment of microbes is required to draw a universal conclusion.

**Fig. 7:**
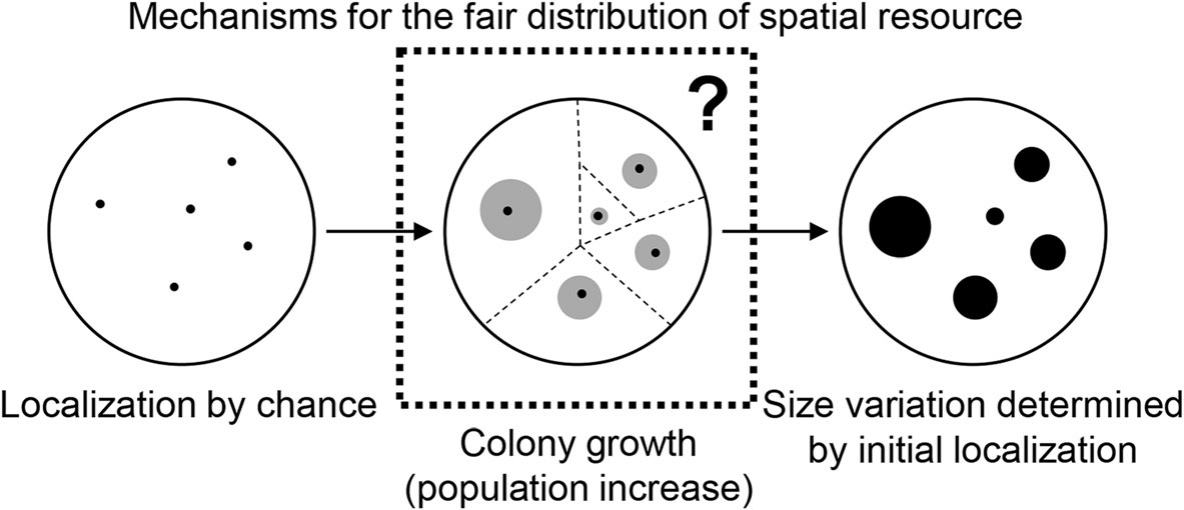
Schematic drawing of the colony localization-determined size variation. The fair distribution of spatial resource based on the colony size determined by chance was proposed. The initial localization of the colonies on the plate (left) determines the final sizes of the colonies (right), which occupy the space areas positively correlated with their final sizes (middle); however, the mechanisms underlying the fair distribution of the space are unclear

The localization-dependent colony growth revealed a common rule for the spatial area occupied (*Voronoi* area) by the colonies, that is, the localization of the first cell on the agar plate, which was occasionally decided by plating, had a deterministic effect on the final colony size. This may sound trivial to a microbiologist, but no quantitative demonstration had been performed until a previous study first reported the *Voronoi* diagram as an available tool for the spatial analysis of microbial colonies on agar plates [17]. The present study further demonstrated that the *Voronoi* diagram is a universal tool for colony analysis and found for the first time a common positive correlation between colony size and the *Voronoi* area. The initial localization decided by chance determined the maximal population size for the geographically separated populations, leading to the variation in population size (Fig. 7). Although both the genomic and nutritional changes disturbed the final population size and the size variation, the rule for the fair distribution of the spatial resource remained constant. This universal phenomenon could be simply considered the fair distribution of nutritional resources for colony/population growth, but the underlying rule, either a chemical or physical mechanism, remains unclear.

Experimental ecology has proposed for centuries [28] as an approach to achieve an improved understanding of ecosystems in the wild, which are highly complex and difficult to control. A well-defined and simplified microbial ecosystem for use in the laboratory could allow us to observe living microorganisms in a quantitative and precisely controllable manner, making it possible to discover the common features and underlying working principles in these ecosystems. The present study successfully found a common feature of the spatial distribution-correlated population increase (colony growth) in the laboratory bacterial strain of *E. coli*, which provided a simple demonstration for connecting the geographically separated monogenetic populations to the space resource. High-throughput experimental tests and analysis of different bacterial species, in particular, of those environmental microbes, are highly required, to draw a universal conclusion on the common rules participated in the spatial distribution of colonies.

## Conclusions

The present study investigated the size variation of colonies of microorganisms (*E. coli*) with different genomes and well-defined culture conditions at different nutritional levels. Although the colonies were randomly located on the agar plates, the colonies at the steady phase were proportional to a geometric quantity, *Voronoi* area, therefore, that in turn is determined only by their initial localization decided by chance. To explain the common correlation, the hypothesis on the localization dependence of colony size for the fair distribution of spatial resources was proposed. The present study provided a polit example to quantitatively explore the mechanisms and/or principles governed in microbial life by means of experimental ecology.

## Methods

### *E. coli* strains

A total of seven *E. coli* strains were used, including the wild-type, genome-reduced and genomic recombinant strains. The analyses were mainly performed on two representative *E. coli* strains, i.e., the wild-type strain K-12 W3110 and its derivative genome-reduced strain, in which 22% of the genomic sequence was deleted [24]. In addition, five different genome-reduced strains (i.e., the *E. coli* K-12 MDS42 series, which contained a foreign gene cassette [29]) were used to verify the universal correlation between the size of the colony and the space occupied by the colony.

### Culture and imaging

Glycerol stocks of the *E. coli* cell cultures, which were prepared beforehand [30], were diluted to final concentrations of 500 ~ 1000 cells/mL in test tubes. Dilution was performed with either the rich medium LB (Luria-Bertani, Sigma) or the minimal medium M63 [30], corresponding to the agar plates used for analysing colony growth. Then, 100 μL of each diluted cell culture was plated on agar plates (1.5% agar), and the plates were incubated at 37 °C in an incubator (THS030PA, ADVANTEC). A total of nine replicates were performed for each condition, and colony growth on the agar plate was imaged by CCD (charge-coupled device) photography. The agar plates were photographed with a high-sensitivity monochrome CCD camera of a gel imager (AE-6932GXES print graph, ATTO Co., Ltd.). The brightness, contrast, saturation, hue and sharpness were set at 50, 73, 50, 50 and 0%, respectively. The OSD (on-screen display) time and exposure time were set at 10 s and 1 s, respectively. Temporal changes in colony growth were observed at 12, 18, 24, 36 and 48 h for the LB medium and 24, 36, 48, 60, 72, 84, 96 and 108 h for the M63 medium. The images were saved as TIF files and subjected to the computational analysis described below. A total of 242 plates and 638 images (photos) were analysed in the present study.

### Colony count and size calculation

Image data analysis was performed with the open source image processing package Fiji based on ImageJ (https://imagej.nih.gov/ij/). Background subtraction and binarization of the image data were performed. The edges of the plates were removed from the binarized images to prevent noise from disturbing the subsequent analysis. The number of colonies formed on the plate (colony count), the relative size of the colony (area in pixels), the relative positions of the colonies on the plate, and the centre position of the plate were determined automatically with an original Java script add-in. The accuracy of the automatic colony counting was confirmed. The coordinates of the colonies and the centre of the plate were exported for the subsequent spatial analysis.

### Central distance analysis

The relationship between the size of the colonies and the distance from the colony to the centre of the plate was investigated. The distance from the centre of the agar plate to the colony (*D*_*j*_) was calculated according to the following formula (Eq. 1).

1 $$ {D}_j=\sqrt{{\left({x}_j-{\mathrm{a}}_i\right)}^2+{\left({y}_j-{b}_i\right)}^2} $$

Here, *x*_*j*_ and *y*_*j*_ represent the X and Y coordinates of colony *j* on the plate *i*; *a*_*i*_ and *b*_*i*_ indicate the coordinates of the centre point of the plate *i*. The coordinates of both the colonies on the plate and the centre of the plate were acquired from the imaging analysis described above.

### Voronoi diagram analysis

The *Voronoi* diagram, which was proposed a century ago [31], was employed for determining the colony distribution on the agar plate. It is a representative approach used in spatial analysis [25] to divide a plurality of points (generic points) localized in a certain metric space into individual regions, named the *Voronoi* areas. The analysis was performed according to the following equations (Eqs. 2.1–2.4).

2.1 $$ C=\left\{{c}_1,{c}_2,\dots, {c}_n\right\} $$

2.2 $$ V\left({c}_l\right)=\left\{c\ \right|\ d\left(c,{c}_l\right)\le d\left(c,{c}_m\right),m\ne l\Big\} $$

2.3 $$ d\left(c,{c}_l\right)=\sqrt{{\left(x-{x}_l\right)}^2+{\left(y-{y}_l\right)}^2} $$

2.4 $$ d\left(c,{c}_m\right)=\sqrt{{\left(x-{x}_m\right)}^2+{\left(y-{y}_m\right)}^2} $$

Here, *V*(*c*_*l*_) and *d* represent the *Voronoi* area of the colony *c*_*l*_ and the function of the distance, respectively. *x* and *y* represent the X and Y coordinates. *l* and *m* are natural numbers less than or equal to *k*, the number of colonies. The colonies and the agar plate were considered as the genetic points (*c*_*1*_, *c*_*2*_, …, *c*_*n*_) and the metric space (*C*), respectively. The coordinates of the colonies on the plate were used for the *Voronoi* division. The tessellation {*V*(*c*_1_), *V*(*c*_2_), …, *V*(*c*_*k*_)} for each plate refers to the *Voronoi* diagram. The *Voronoi* areas were determined with the function ‘*dirichlet*’ in the spatial statistics package *spatstat*, which was implemented in the programming software R [32]. The computational analysis and graphics preparation were performed with R. The resultant data could be found in Tables S1 and S2.

## Supplementary Information

**Additional file 1: Table S1**. Data used for the boxplots. **Table S2**. Data of temporal changes in colony size and Voronoi correlation.

## Data Availability

Not applicable.
